# Opioid Medical Detoxification Compared to Opioid Agonist Treatment during Pregnancy: A Scoping Review

**DOI:** 10.3390/healthcare12131270

**Published:** 2024-06-26

**Authors:** Alice Ordean, Isabella DeVuono

**Affiliations:** 1 Department of Family Medicine, St. Joseph’s Health Centre, Unity Health Toronto, Toronto, ON M6R 1B5, Canada; 2 Li Ka Shing Knowledge Institute, Unity Health Toronto, Toronto, ON M6R 1B5, Canada; 3 Department of Family and Community Medicine, University of Toronto, Toronto, ON M5G 1V7, Canada; 4 Institute of Medical Science, University of Toronto, Toronto, ON M5S 1A8, Canada; isabella.devuono@mail.utoronto.ca

**Keywords:** opioid use disorder, pregnancy, opioid agonist treatment, opioid detoxification

## Abstract

Opioid use disorder (OUD) is highly prevalent, affecting up to 1% of pregnancies. The current standard of care for the management of OUD during pregnancy has been maintained with opioid agonist treatment (OAT), using either methadone or buprenorphine. OAT use has been associated with a risk of neonatal abstinence syndrome (NAS), which requires a longer neonatal length of stay for monitoring and possible pharmacological treatment. As a result, opioid medical detoxification (OMD) was proposed as an alternative strategy to reduce the stigma associated with OAT and to eliminate the risk of NAS by detoxifying or tapering pregnant persons during their pregnancy before delivery; however, the safety and effectiveness of OMD during pregnancy have not been established. This scoping review aims to summarize recent evidence related to maternal, obstetrical, and neonatal outcomes of OMD in comparison to OAT maintenance. This review also provides recommendations for future research initiatives to fill gaps in managing this patient population.

## 1. Introduction

Opioid use disorder (OUD) is a chronic, relapsing condition associated with several negative medical and social consequences [1]. While the risks of OUD are similar for pregnant and non-pregnant populations, there are additional obstetrical complications during pregnancy that must be considered as part of the management plan. When OUD precedes pregnancy, it is associated with adverse outcomes for both pregnant persons and their unborn child(ren). Data from the 2022 National Survey on Drug Use and Health showed that the past month’s prevalence of illicit drug use increased from 5.8% to 9.6% in the period from 2019 to 2022 [2]. More specifically, the estimated rate of past month’s opioid misuse in pregnant persons, including illicit fentanyl, was approximately 1% [2]. Over the past decade, illicit fentanyl and fentanyl analogs have contributed to the development of an unregulated, contaminated, and highly toxic drug supply [3]. The increasing prevalence of illicit opioid use during pregnancy reinforces the need for optimizing OUD management strategies to ensure the safety of the mother-neonatal dyad.

The standard of care for the management of OUD during pregnancy involves the use of opioid agonist treatment (OAT), with methadone or buprenorphine. This treatment approach is endorsed by numerous professional organizations, including the American Society of Addiction Medicine and the Canadian Research Initiative in Substance Matters [1,3]. OAT maintenance consists of providing daily opioid doses that prevent withdrawal symptoms and reduce cravings. Antenatal opioid use including OAT has been associated with transplacental transfer of opioids from the pregnant person to the fetus resulting in pregnancy complications, such as intrauterine growth restriction, preterm birth, and even intrauterine fetal demise [3]. Postnatally, neonatal abstinence syndrome (NAS), now termed neonatal opioid withdrawal syndrome (NOWS), is a common consequence of antenatal opioid exposure and requires prolonged neonatal length of hospital stay for monitoring and possible pharmacological treatment of neonatal withdrawal [1,3]. A growing number of studies comparing outcomes of methadone to buprenorphine use during pregnancy indicate that buprenorphine may lead to reduced NAS severity without any significant differences in maternal outcomes; however, larger randomized studies are needed to provide concrete evidence regarding the safety and efficacy of these two agents for pregnant persons [3].

Opioid medical detoxification (OMD) involves a gradual medication-assisted withdrawal using opioid agonist agents, such as methadone or buprenorphine instead of continued maintenance for the duration of the pregnancy. Consequently, pregnant persons may consider OMD as an alternative management strategy to reduce the stigma associated with OAT use during pregnancy, especially to avoid NAS. Historically, OMD was not recommended during pregnancy due to theoretical risks of increased adverse maternal, obstetrical, and neonatal outcomes [1]. Although OMD may eliminate the risk of NAS, it has been debated whether the process of OMD increases the risk of other negative outcomes during the perinatal period.

Two prior systemic reviews on this topic have been published with some methodological limitations [4,5]. Terplan et al. included 15 studies published between 1966 and 2016, of which seven were conducted prior to the Food and Drug Administration approval of buprenorphine in 2002, as well as older studies from the 1960s where sudden cessation of opioids was reported [4]. In addition, some studies used clonidine or rapid detoxification with sedation as detoxification methods, both of which are not recommended for the management of OUD during pregnancy [4]. Wang et al. included only three studies published from 2008 to 2014, and two of these consisted of persons from the same treatment center, which may have resulted in duplicate reporting of findings on overlapping populations [5]. These methodological concerns represent some of the weaknesses of these previously published reviews. Therefore, the objective of this scoping review was to provide an evidence-based analysis regarding the safety and efficacy of OMD in comparison to OAT maintenance. This review also aimed to identify knowledge gaps in the current literature for the optimal management of OUD during pregnancy and to provide recommendations for future research initiatives.

## 2. Materials and Methods

This scoping review is reported in accordance with the Preferred Reporting Items for Systematic Reviews and Meta-Analyses extension for Scoping Reviews [6].

### 2.1. Eligibility Criteria

#### 2.1.1. Inclusion Criteria

This review included primary outcome studies of pregnant persons with OUD defined as misuse of prescribed, diverted, or illicit opioids that were offered OMD without requiring transition to long-term OAT. We included peer-reviewed studies involving human subjects, published in the English language from January 2000 to August 2022. Studies were included if they provided data on maternal, obstetrical, or neonatal outcomes of interest.

#### 2.1.2. Exclusion Criteria

Non-empirical study designs including reviews, meta-analyses, protocols, abstracts, conference papers, book chapters, thesis dissertations, case reports, case series, editorials, commentaries, and non-peer-reviewed studies were excluded from this review.

### 2.2. Outcomes

Studies were included if at least two of the following outcomes of interest were reported:

- Maternal substance use and opioid detoxification outcomes;
- Obstetrical outcomes: spontaneous abortion, intrauterine fetal demise, and antenatal hemorrhage;
- Neonatal outcomes: birth parameters; NAS prevalence, severity, and treatment.

### 2.3. Information Sources, Search Strategy, and Study Selection Process

**Figure 1:**
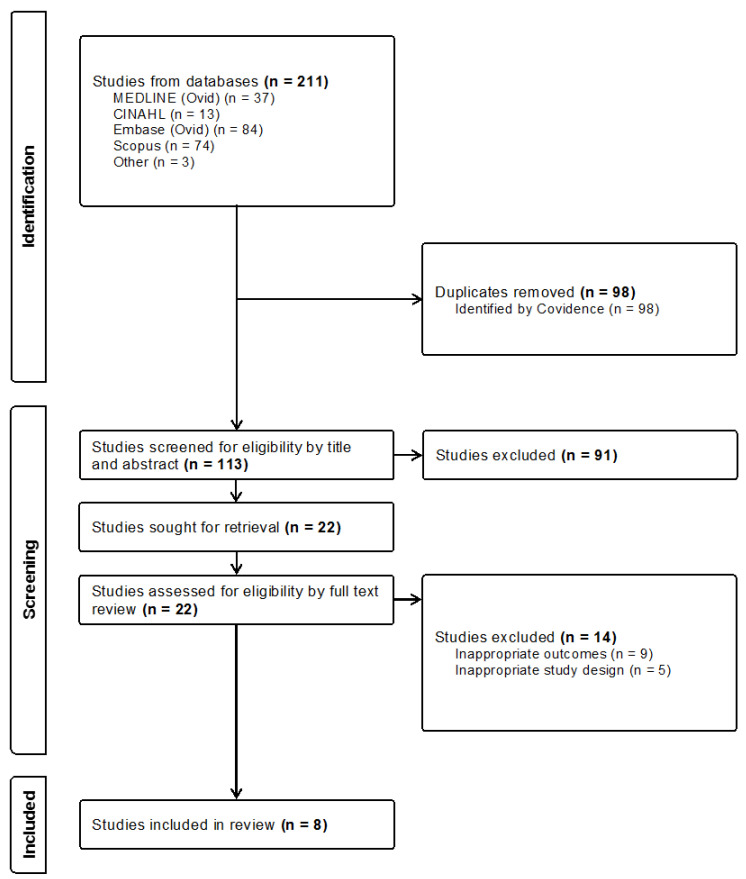
Preferred reporting items for systematic reviews and meta-analyses flow diagram displaying identification of studies via databases and manual search.

The following databases were searched: MEDLINE (via Ovid), CINAHL, Embase (via Ovid), and Scopus using the following Medical Subject Headings: pregnancy, pregnancy outcome(s), pregnancy complication(s), pregnant women/expectant mothers, opioid-related disorders/substance use disorders, and detoxification. The reference lists of studies that met our inclusion criteria were manually reviewed and three additional studies were found. All studies were imported into Covidence for title and abstract screening, full-text review, and data extraction. One of the authors (I.D.) conducted the initial literature search, which identified 113 studies after removing duplicates (Figure 1). Then, both authors independently screened all titles and abstracts for relevance to this review. Finally, both authors screened all 22 full-text studies and found that eight met our inclusion criteria (Figure 1).

### 2.4. Data Extraction and Synthesis

A data extraction table was developed and utilized to review study characteristics (year of publication, country, type of study, and number of participants), maternal demographics of the detoxification group, and detoxification process (type of opioid and duration of detoxification), as well as to collect maternal, pregnancy, and neonatal outcomes. Before the data extraction was initiated, both authors performed an inter-rater reliability test using one of the studies for the calibration of data extraction and agreement among reviewers. Then, both authors completed data extraction for the eight papers that met our inclusion criteria. Any disagreements were discussed until a consensus was reached.

## 3. Results

### 3.1. Study Characteristics

Eight studies met our inclusion criteria (Table 1). These eight studies were published between 2003 and 2020 and were conducted in the United States of America (n = 5) [7,8,9,10,11], Norway (n = 2) [12,13], and the United Kingdom (n = 1) [14]. Studies used a variety of study designs consisting of retrospective (n = 6), prospective (n = 1), and mixed prospective/retrospective (n = 1) cohorts. Four studies utilized comparison groups which consisted of opioid maintenance groups (n = 3) [8,9,13] or non-using control groups (n = 1) [12].

### 3.2. Maternal Outcomes

#### 3.2.1. Maternal Demographics

The eight studies included over 850 pregnant persons who underwent OMD or tapering, using methadone or buprenorphine [7,8,9,10,11,12,13,14]. On average, pregnant persons had a mean age between 25 and 30 years with a mean education level of 11 years (n = 8) [8,9,10,11,13,14]. Further limited demographic data indicated that the majority of women were single (74–95%) [8,9,14], and more than half were multiparous with a range of mean parity from 1 to 3.5 [8,11,13]. Race was reflective of geographical location with studies including mostly white and African American women with other minority groups not significantly represented [7,8,9,12,14].

#### 3.2.2. Opioid Medical Detoxification and Maternal Relapse Rates

OMD varied in each study (Table 1 and Table 2). This process ranged from inpatient admissions using quick detoxification protocols of short duration to slower tapering of methadone or buprenorphine doses over weeks or months in outpatient settings. All studies used standard OAT, which consisted of either methadone (n = 4) [8,11,12,14], buprenorphine (n = 2) [7,10], or a combination of methadone or buprenorphine (n = 2) [9,13] (Table 2). The mean gestational age at the start of OMD was between 20 and 22 weeks with a duration of inpatient opioid detoxification, varying from as short as 3 days or 7 days to 15–25 days [7,8,11,14]. Completion rates for full opioid detoxification ranged from 2 to 100%, whereas slow tapering of opioid doses ranged from 15 to 100% (Table 2).

Maternal relapse rates were not reported consistently across these studies. Bell et al. only included women who were fully detoxified during pregnancy but showed an overall relapse rate of 36% as determined by a positive drug screen on admission or patient self-report of relapse at the time of delivery [7]. In this study, women who were fully detoxified and then followed in outpatient behavioral health programs were significantly less likely to relapse than those who were managed without intense outpatient follow-up. Jones et al. showed that methadone-assisted withdrawal was associated with statistically significant higher rates of positive urine toxicology tests for illicit drugs at delivery (53–57% versus 15–33%) [8]. Also, a longer tapering period was associated with longer treatment retention as indicated by higher rates of transfer to methadone maintenance treatment.

Luty et al. also documented less than 50% completion rate for opioid detoxification and limited follow-up after admission to the inpatient unit, with only 7.9% of the detoxified group being discharged to a residential treatment program [14]. In addition, based on the limited outcome data at delivery, only 5% were found to have remained abstinent from all opioids at delivery including methadone. Similarly, Stewart et al. reported a success rate of 56% for OMD completion and 18% opted to drop out of OMD to initiate OAT maintenance [11]. Those who were successful in remaining opioid free had a statistically significant shorter duration for detox completion (15 days versus 25 days) and had a lower dropout rate (9% versus 33%) [11].

#### 3.2.3. Maternal Illicit Substance Use

Data related to maternal substance use was provided by only five studies [8,10,11,12,13]. Polysubstance use was commonly reported at the first visit including self-reported use of alcohol, tobacco, cannabinoids, amphetamines, and cocaine (Table 3). Studies documented that by delivery, rates of substance use were lower for all substances including alcohol, tobacco, cannabinoids, amphetamines, and opioids. These reductions may be attributed to involvement in care for opioid use disorders and may not be directly related to the management of their opioid use disorder.

### 3.3. Obstetrical Complications

Among the included studies, pregnancy complications were not frequently reported. There were no reported rates of antenatal hemorrhage. Women who underwent OMD were found to have low rates of spontaneous abortion and intrauterine fetal demise [7,8,10,11,14]. Miscarriage rates varied from 1 to 4% with a higher rate among pregnant persons who underwent a seven-day OMD [7,8,14]. Similarly, intrauterine fetal demise was reported by up to 5% of the population, especially with concomitant use of illicit substances at delivery [7,10,14]. These fetal loss rates were not deemed to be above general population means; thus, complications were found to be low among the different cohorts of pregnant persons.

### 3.4. Neonatal Outcomes

#### 3.4.1. Birth Parameters

Most studies did not report the type of delivery. Only one study specifically mentioned the rate of spontaneous vaginal delivery was between 75% and 90% [13]. Mean birth parameters were also not uniformly reported (Table 4). The mean birth weight, length, and head circumference were between 2564 and 3245 g, 48 cm, and 32 cm, respectively [8,10,11,12,13,14]. The mean gestational age at delivery indicated that most neonates were born at term between 37 and 40 weeks.

#### 3.4.2. Neonatal Abstinence Syndrome (NAS)

NAS is a complex disorder which presents as a postnatal withdrawal syndrome secondary to in utero substance exposure [15]. While NAS is considered an expected and treatable outcome of maternal opioid use, its severity cannot be predicted. NAS presentation can be modified by maternal, infant, and environmental factors [15,16]. In our review, none of the included studies provided any details related to NAS assessment and management protocols [7,8,9,10,11,12,13,14]. NAS prevalence rates and treatment rates were only reported by four studies due to the differing maternal detoxification protocols and showed a wide variability in findings (Table 4) [7,9,10,12]. Not surprisingly, cohorts that reported 100% successful detoxification reported no cases of NAS [9,10,12]. In other studies where pregnant persons did not complete detoxification or underwent short detoxification protocols, the rates of NAS were up to 80% among neonates requiring pharmacotherapy, especially for those exposed to illicit drug use at delivery. Similarly, the length of pharmacological treatment was not reported by any of these studies. However, the mean neonatal length of hospital stay (LOS), as reported by three studies, ranged from 3 to 33 days [8,11,13]. Stewart et al. found that the mean LOS was longer for those neonates whose mothers were documented to have had illicit substance use prior to delivery [11]. This finding is consistent with previous evidence that endorsed that in general, polysubstance exposure can increase the severity of NAS expression [15]. Beyond pharmacological management, there were no data reported on non-pharmacological interventions such as rooming-in and care by parents, breastfeeding, and other supportive measures [7,8,9,10,11,12,13,14]. These factors are important determinants of the severity of NAS and were not addressed by these studies [15,16,17].

## 4. Discussion

This review consisted of studies that addressed OMD as an alternative strategy to opioid agonist maintenance treatment for the management of OUD during pregnancy. Based on the studies included, OMD represented an umbrella term representing two different options: rapid opioid-assisted detoxification of short duration lasting from 3 to 7 days up to 25 days in contrast to slower tapering or dose reductions using OAT such as methadone or buprenorphine over weeks to months. Studies also differed in terms of whether women were already on OAT prior to initiation of detoxification or tapering protocols [10,13]. No consistent detoxification or tapering process was utilized, thereby making it difficult to compare findings across studies.

Overall detoxification rates were low across most studies. When reviewing findings based on the type of OMD, studies that used an inpatient rapid detoxification protocol demonstrated approximately 50% completion rates, with a significant proportion (10.6% to 41.6%) opting to switch to methadone or buprenorphine maintenance treatment [8,11,14]. These studies also demonstrated high relapse rates to opioids and other illicit substance use by delivery. In contrast, studies that addressed tapering off OAT over weeks to months demonstrated a wider range of outcomes including full detoxification and dose reductions by delivery [9,10,13]. Olsen et al. reported a 100% detoxification success rate using a slow outpatient buprenorphine tapering protocol for women who were predominantly on buprenorphine at the time of the first visit [10]. In contrast, Welle-Strand et al. found that only 2.7% completed detoxification of OAT, with 68% reducing OAT dose by delivery [13]. It is also important to note that maternal polysubstance use was highly prevalent during the first visit and was significantly reduced by delivery despite attempts at OMD.

Beyond maternal substance use and relapse rates, obstetrical and neonatal consequences related to OMD need to be considered. Obstetrical outcomes were not well documented; however, spontaneous abortion and intrauterine fetal demise were rare occurrences [7,8,10,11,12,14]. Due to the paucity of such reports, no conclusive statements can be made regarding pregnancy-related complications secondary to OMD.

The impact of OMD on neonatal birth parameters and NAS were also highly variable in our included studies. Data on birth parameters consisted mostly of birth weight and gestational age at delivery. The mean birth weight ranged from 2564 to 3245 g with an average gestational age of 37.5 to 39.6 weeks at delivery [8,10,11,12,13,14]. Furthermore, infants born to pregnant persons who successfully completed the detoxification process did not experience any withdrawal [9,10,12]. Based on outcomes from studies where detoxification was not successful, the prevalence and treatment of NAS were related to persistent or relapse to illicit substance use by delivery [8,11,14]. Since completion of detoxification did not result in the complete elimination of NAS among some cohorts, it is probable that some of the women who detoxified at an earlier point in their pregnancies ended up relapsing or resuming opioid and other illicit substance use by delivery. Three studies also suggested that tapering the dose of OAT contributed to lower prevalence and/or treatment rates for NAS which is contradictory to past reviews which did not support any association between maternal OAT dose and severity of NAS [7,9,13]. These findings based on small sample sizes need to be interpreted with caution since the decision to taper OAT during pregnancy should be based on the person’s substance use stability and medical status.

There are several limitations to this review. First, the studies related to OMD were heterogeneous in terms of methodology for maternal detoxification and reporting of associated findings. Since there is no accepted standard for maternal opioid detoxification, some researchers implemented rapid detox protocols, which may not be considered a routine part of clinical practice. Furthermore, there was limited data provided about the characteristics of participants making it difficult to determine the applicability of these approaches to different patient populations. Finally, details related to NAS assessment and management were not provided by most studies, making it difficult to interpret NAS outcomes in individuals maintained on OAT in comparison to those undergoing opioid detoxification. Given that pregnant persons indicated avoidance of NAS as one of the driving factors for considering medical detoxification, the lack of details related to NAS outcomes among included studies is a significant limitation and as such, caution must be used when applying findings related to maternal opioid detoxification in practice. A recent systematic review and meta-analysis indicated that non-pharmacological interventions such as rooming-in may be effective at reducing the severity of NAS leading to reduced prevalence of pharmacotherapy for NAS and enhanced breastfeeding rates [15,17].

Since OMD consisting of rapid detoxification did not lead to favorable maternal and neonatal outcomes, our review supports recommendations for OAT maintenance over maternal opioid detoxification. Similarly, tapering off OAT more gradually did not eliminate the risk of NAS while placing the fetus at risk for intrauterine withdrawal, which also makes OAT maintenance the preferred management approach for OUD during pregnancy. Pregnant persons should be counseled on the maternal, obstetrical, and neonatal risks of OUD, OAT maintenance, and OAT detoxification or dose tapering. Based on this review, for a subset of pregnant persons who request OMD, a slow and gradual methadone or buprenorphine taper is preferable to sudden discontinuation or rapid detoxification protocol to reduce maternal, obstetrical, and neonatal outcomes. Shared decision-making should be grounded in up-to-date and evidence-based approaches to the management of OUD during pregnancy to ensure the safety of both the mother and child.

## 5. Conclusions

The safety and effectiveness of OMD during pregnancy have been controversial. While OMD has the potential to eliminate or reduce the risk of NAS, this risk should be balanced with maternal relapse to substance use, which may have other adverse neonatal outcomes. A comprehensive discussion of the benefits and risks of different management options is highly recommended to create a tailored approach to the management of OUD during pregnancy. Further high-quality studies are required to address this clinical debate.

## Figures and Tables

**Table 1:** Description of included studies (n = 8).

First Author, Year (Country)	Opioid Detoxification Protocol	Intervention Group	Comparison Group
Bell et al., 2016 [7] (United States of America)	Four different detoxification groups Group 1: involuntary acute detoxification Groups 2 and 3: inpatient buprenorphine detoxification in 5 to 8 days Group 4: outpatient slow buprenorphine detoxification over 8 to 16 weeks	n_1_ = 108 n_2_ = 23 n_3_ = 77 n_4_ = 93	Not applicable
Haabrekke et al., 2014 [12] (Norway)	Four cohorts from two different time periods Cohort 1: outpatient opioid maintenance Cohort 2: inpatient opioid detoxification	n_1_ = 78 n_2_ = 31	Non-using population n_1_ = 58 n_2_ = 30
Jones et al., 2008 [8] (United States of America)	Inpatient 3-day or 7-day methadone assisted withdrawal ± methadone maintenance	n_1_ = 95 withdrawal n_2_ = 28 withdrawal, and then MMT	n = 52 MMT
Luty et al., 2003 [14] (United Kingdom)	Inpatient 21-day methadone detoxification	n = 101	Not applicable
Macfie et al., 2020 [9] (United States of America)	Outpatient slow detoxification over weeks to months with methadone or buprenorphine	n = 32 13 taper and 19 detoxifications	n = 23 MMT or BMT
Olsen, 2020 [10] (United States of America)	Outpatient buprenorphine taper over weeks to months Dose reduced by 2 mg every 4 weeks	n = 21	Not applicable
Stewart et al., 2013 [11] (United States of America)	Inpatient methadone detoxification Dose reduced by 20% every 1 to 3 days	n = 95	Not applicable
Welle-Strand et al., 2015 [13] (Norway)	Outpatient tapering of methadone or buprenorphine maintenance treatment Group 1: dose reduced >50% Group 2: dose reduced 11–50% Group 3: stable or increased dose	n = 75 attempted taper	n = 48 did not taper MMT or BMT

MMT: methadone maintenance treatment; BMT: buprenorphine maintenance treatment.

**Table 2:** Opioid medical detoxification outcomes.

First Author	OAT Used	Duration and Timing of OMD	OMD Outcomes
Bell et al., 2016 [7] (United States of America)	Buprenorphine	Duration groups 2 and 3: 5 to 8 days Duration group 4: 8 to 16 weeks 28 detoxed in T1 148 detoxed in T2 125 detoxed in T3	100% completed detoxification 36% relapse rate (all groups) (range 17.4–74.0%) *
Haabrekke et al., 2014 [12] (Norway)	Methadone	4 detoxed in T1 7 detoxed in T2 10 detoxed in T3	0% dropped out or relapsed from inpatient detoxification program
Jones et al., 2008 [8] (United States of America)	Methadone	Onset at GA (mean): 20.3 weeks Duration of detoxification: 3 or 7 days	41.6% of 7-day detoxification versus 10.6% of 3-day detoxification transferred to MMT Withdrawal groups had higher positive urine toxicology tests at delivery *
Luty et al., 2003 [14] (United Kingdom)	Methadone	Onset at GA (mean): 22.5 weeks Duration of detoxification: 19.5 days	42% completed detoxification 56% follow-up rate after detoxification 95% relapse rate by delivery
Macfie et al., 2020 [9] (United States of America)	Methadone, buprenorphine, or buprenorphine-naloxone	Data not provided	45% completed detoxification 24% of the detoxification group switched to MAT by delivery 0% dropped out 26% of MAT versus 0% of detoxification groups relapsed **
Olsen, 2020 [10] (United States of America)	Buprenorphine	Duration of detoxification: minimum of 16 weeks with 4-week intervals in dose reductions	100% completed detoxification 48% self-detoxed
Stewart et al., 2013 [11] (United States of America)	Methadone	Onset at GA (mean): 20.1 weeks Duration of detoxification: 15–25 days * based on relapse to drug use by delivery	56% completed detoxification 18% switched to MAT 20% dropped out
Welle-Strand et al., 2015 [13] (Norway)	Methadone or buprenorphine	Duration of detoxification (mean): 60–160 days	61% attempted to taper the dose 68% of those who attempted taper were able to reduce the dose 2.7% completed detoxification No tendency to relapse to opioid use based on dose reduction

T1: first trimester; T2: second trimester; T3: third trimester; GA: gestational age; MAT: medication-assisted treatment; MMT: methadone maintenance treatment. * significant difference at *p* < 0.001; ** significant difference at *p* < 0.05.

**Table 3:** Prevalence of maternal substance use among persons undergoing opioid medical detoxification.

Substance	First Visit (%)	Delivery (%)
Alcohol	11–37%	5–30%
Cannabinoids	14–64%	0–62%
Amphetamines	17–59%	0–17%
Cocaine	50–61%	Not available
Tobacco	79–100%	60–86%

**Table 4:** Neonatal outcomes including birth parameters and neonatal abstinence syndrome.

First Author	Mean Birth Parameters	NAS Prevalence and Treatment Rates
Bell et al., 2016 [7] (United States of America)	No data provided	Prevalence varied by group and ranged from 17.2% to 70.1% Group 1: 18.5% (fully detoxified while incarcerated) Groups 2 and 4: 17.2% (fully detoxified and intense follow-up) Group 3: 70.1% (fully detoxified but no follow-up) * Treatment for NAS: 31% (all groups)
Haabrekke et al., 2014 [12] (Norway)	BW: 3022 g HC: 33.9 cm GA: 38.3 weeks	Prevalence: 0% (cohort 2, fully detoxified group)
Jones et al., 2008 [8] (United States of America)	BW: 2853 g HC: 32.3 cm Length: 48.1 cm GA: 37.7 weeks	Treatment for NAS: 26% (all groups)—no significant difference between methadone-assisted withdrawal in comparison to methadone maintenance groups
Luty et al., 2003 [14] (United Kingdom)	BW: 2564 g GA: 37.5 weeks	No data provided
Macfie et al., 2020 [9] (United States of America)	No data provided	Prevalence varied by group Prevalence: 0% detox group versus 62% taper group Prevalence: 91% MAT group No data provided on treatment for NAS
Olsen, 2020 [10] (United States of America)	BW: 3080 g GA: 39.1 weeks	Prevalence: 0% (fully detoxified group)
Stewart et al., 2013 [11] (United States of America)	BW: 3065 g GA: 39 weeks	Treatment for NAS related to illicit substance use at delivery Max NAS score, number needing treatment for NAS and LOS were statistically different between 2 groups * 10% versus 80% treatment rates for NAS
Welle-Strand et al., 2015 [13] (Norway)	BW: 3245 g HC: 34.0 cm Length: 48.6 cm GA: 39.6 weeks	Treatment rates for NAS and length of stay varied by group Group 1: 38% treated for NAS Group 2: 63% treated for NAS Group 3: 62% treated for NAS No significant differences between groups

BW: mean birth weight; cm: centimeters; g: grams; GA: mean gestational age at birth; HC: mean head circumference; NAS: neonatal abstinence syndrome; * statistically significant at *p* < 0.0001.
